# Learning Latent Space Representations to Predict Patient Outcomes: Model Development and Validation

**DOI:** 10.2196/16374

**Published:** 2020-03-23

**Authors:** Subendhu Rongali, Adam J Rose, David D McManus, Adarsha S Bajracharya, Alok Kapoor, Edgard Granillo, Hong Yu

**Affiliations:** 1 College of Information and Computer Sciences University of Massachusetts Amherst Amherst, MA United States; 2 Section of General Internal Medicine Boston University School of Medicine Boston, MA United States; 3 Department of Medicine University of Massachusetts Medical School Worcester, MA United States; 4 Meyers Primary Care Institute Worcester, MA United States; 5 Department of Population and Quantitative Health Sciences University of Massachusetts Medical School Worcester, MA United States; 6 Department of Computer Science University of Massachusetts Lowell Lowell, MA United States; 7 Center for Healthcare Organization and Implementation Research Bedford Veterans Affairs Medical Center Bedford, MA United States

**Keywords:** predictive modeling, neural networks, ablation, patient mortality

## Abstract

**Background:**

Scalable and accurate health outcome prediction using electronic health record (EHR) data has gained much attention in research recently. Previous machine learning models mostly ignore relations between different types of clinical data (ie, laboratory components, International Classification of Diseases codes, and medications).

**Objective:**

This study aimed to model such relations and build predictive models using the EHR data from intensive care units. We developed innovative neural network models and compared them with the widely used logistic regression model and other state-of-the-art neural network models to predict the patient’s mortality using their longitudinal EHR data.

**Methods:**

We built a set of neural network models that we collectively called as long short-term memory (LSTM) outcome prediction using comprehensive feature relations or in short, CLOUT. Our CLOUT models use a correlational neural network model to identify a latent space representation between different types of discrete clinical features during a patient’s encounter and integrate the latent representation into an LSTM-based predictive model framework. In addition, we designed an ablation experiment to identify risk factors from our CLOUT models. Using physicians’ input as the gold standard, we compared the risk factors identified by both CLOUT and logistic regression models.

**Results:**

Experiments on the Medical Information Mart for Intensive Care-III dataset (selected patient population: 7537) show that CLOUT (area under the receiver operating characteristic curve=0.89) has surpassed logistic regression (0.82) and other baseline NN models (<0.86). In addition, physicians’ agreement with the CLOUT-derived risk factor rankings was statistically significantly higher than the agreement with the logistic regression model.

**Conclusions:**

Our results support the applicability of CLOUT for real-world clinical use in identifying patients at high risk of mortality.

## Introduction

### Background

High-precision predictive modeling of clinical outcomes (eg, adverse events such as the onset of disease and death) is a clinically important but computationally challenging task. If physicians can be notified about the risks of adverse events in advance, they may be able to take steps to prevent them. Electronic health records (EHRs) are widely used in US hospitals and are becoming more mature over time [1]. They have been actively researched for predictive modeling [2-8].

Almost 6 million patients are admitted annually to intensive care units (ICUs) in the United States for airway support, for hemodynamic or respiratory monitoring, and to stabilize acute or life-threatening medical problems [9-15]. Patients in ICUs are vulnerable to many acute diseases and often suffer from chronic illness, but the leading causes of death in the ICU are multi-organ failure, sepsis, and cardiovascular disease. Approximately 10% to 30% of adult patients die before hospital discharge in ICUs [16-30]. Regression models have been widely used for predicting mortality for ICU patients [31]. Goal-directed sepsis care represents an example of a successful, evidence-based approach to the care of critically ill patients with sepsis that uses predictive modeling to target patients at high risk for mortality with life-saving upstream therapies [21].

During the past several years, neural network models have shown a great success for many artificial intelligence applications including computer vision, natural language processing, and predictive modeling [4,32-34]. Neural network-based predictive models include the convolutional neural network (CNN) and recurrent neural network (RNN) framework.

Although studies show that CNN models do not necessarily outperform conventional predictive models such as regression models [35], RNNs [36] have been shown to work well with sequential data such as longitudinal EHRs. There have been promising results regarding the use of RNNs in clinical applications such as diagnosis predictions [6,37,38]. Autoencoders [39] are another class of neural networks that extract rich representations using large unlabeled EHR data and have shown state-of-the-art performance in prediction [4].

Although NN-based predictive models have been developed, most models are based on *bag of features*, and few have explicitly modeled the complex relationships between different types of EHR data. Clinical events and diagnoses are not isolated but instead are complex, multifaceted, and often correlated. For example, diagnostic testing leads to a new finding, which may lead to a specific treatment. Therefore, we believe it is important to account for such relationships to improve the predictive power of a model.

### Objective

The main objective of this work is to develop innovative prediction models to accurately predict patient mortality using patients’ longitudinal EHR data. An important component of our models is a correlational neural network, which is a special neural network model that accounts for correlations between different types of features. We modeled the relationships between different types of clinical features in the EHR through a correlational neural network and integrated them into LSTM-based predictive models for improved performance.

### Contributions

Our main contributions include learning of latent features from different clinical data types and integrating the learned latent features for outcome prediction using longitudinal EHR data. Our results show that the integration of latent features yielded the highest results for predicting patient mortality using the ICU data.

In addition to evaluating our CLOUT models using the traditional evaluation metrics such as sensitivity, specificity, and area under the receiver operating characteristic curve, we studied the interpretability of our predictive models. Specifically, we designed a simple ablation experiment [40] to identify important features (or risk factors). Our evaluation results show that physicians were more in agreement with the risk factors ranked by CLOUT than the ones ranked by the commonly used logistic regression model.

In summary, our contributions are twofold: (1) We developed an innovative long short-term memory (LSTM)–based predictive model where a correlational neural network is integrated to identify relationships and latent representations of different clinical features. Our CLOUT model has state-of-the-art performance in mortality prediction, surpassing other competitive NN models and a logistic regression model. (2) We provide a comprehensive evaluation of risk factors identified by our neural network models. Our results show that the risk factors identified by the CLOUT model agree with physicians’ assessment, suggesting that CLOUT could be used in real-world clinical settings.

## Methods

### The Medical Information Mart for Intensive Care-III Dataset

All models are trained and evaluated on the Medical Information Mart for Intensive Care-III (MIMIC-III) dataset; an EHR dataset made publicly available by the Massachusetts Institute of Technology Laboratory for Computational Physiology. MIMIC-III has been widely used for predictive models [41]. The dataset contains 7537 patients with two or more encounters, which is the subset we used to build our CLOUT and baseline models. We call this dataset p-MIMIC. Some demographic information for patients in this dataset is given in Table 1.

**Table 1:** Patient demographic information (N=7537).

Characteristic		Values
**Age (years)**		
	Mean	74.74
	Median	66.00
**Sex, n (%)**		
	Male	4190 (55.59)
	Female	3347 (44.41)
**Race, n (%)**		
	White	5644 (74.88)
	Black	867 (11.50)
	Hispanic	277 (3.68)
	Asian	226 (3.00)
	Other/unknown	523 (6.94)

We require two or more encounters because we remove the last encounter while making predictions, requiring us to have at least one other encounter with data. We use patient mortality as our outcome label. This label is obtained in the MIMIC dataset from the hospital records and the social security death records. In our dataset of 7537 patients, we have 2825 (37.9%) documented deaths. Further details about MIMIC are covered in Multimedia Appendix 1.

The dataset was further divided into train, validation, and test splits, each containing approximately 69.99% (5275/7537), 9.99% (753/7537), and 20.02% (1509/7537) of the patients, respectively. Once we picked the optimal model hyper-parameters using the validation set, the model was retrained on the combined train-validation set, which contained 79.98% (6028/7537) of the data.

### Baselines—Reverse Time Attention Model, Time-Aware Reverse Time Attention Model, Logistic Regression Models

Our first set of baseline models are versions of the *RETAIN* model, which is one of the few publicly available predictive models for EHRs. RETAIN was built on an RNN model, and evaluation has shown that it achieved both state-of-the-art performance and interpretability [6].

RETAIN by itself does not incorporate temporal information beyond the RNN framework; such fine-grained temporal information may be important to patient outcomes. For example, the severity of 2 acute myocardial infarctions separated by different durations could have different clinical implications. On the other hand, there is an option to include the time features to the encounter vector. Therefore, we implemented time-aware RETAIN (TaRETAIN) models as additional baselines by concatenating time information to the input features. We experimented with two different approaches to create the time feature: number of days elapsed since the first encounter and number of days elapsed since the previous encounter. We call these 2 models *TaRETAIN-first* and *TaRETAIN-previous*.

Another baseline model is *logistic regression* as it has been commonly used with EHR data. Although logistic regression is best in interpretability, it is difficult to incorporate temporal information. We therefore combined all the information documented in an encounter to form 1 feature vector for each patient. Our logistic regression model was also augmented with the l2 penalty.

### The CLOUT Models

The CLOUT models are built upon the state-of-the-art LSTM framework. We provide a description of relevant concepts or components that are built into our CLOUT models in Multimedia Appendix 2.

**Figure 1:**
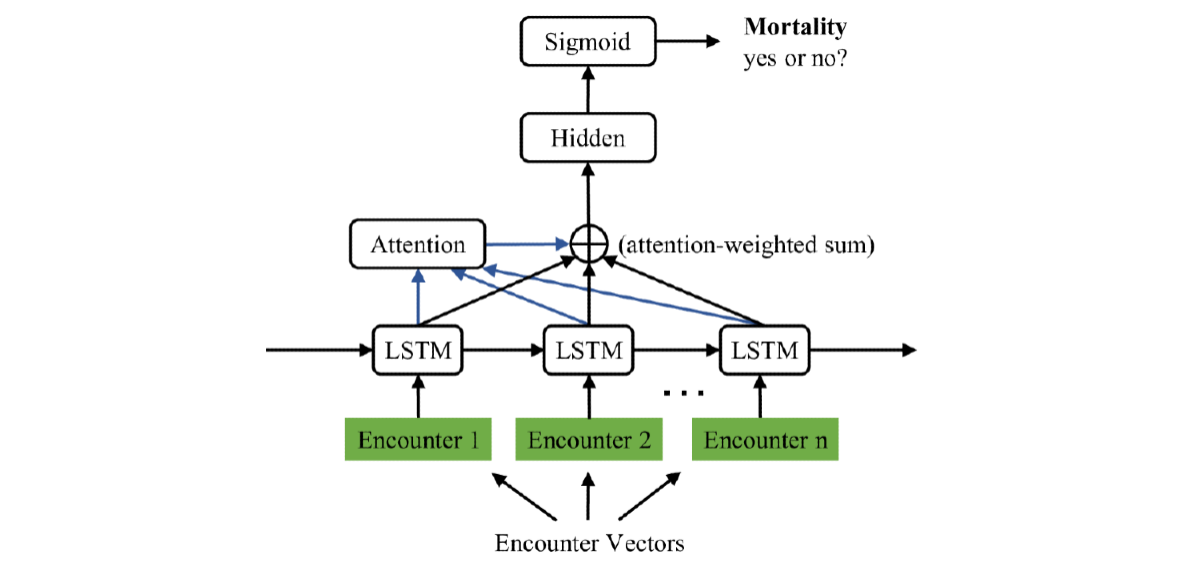
Our model architecture. LSTM: long short-term memory.

Unlike other RNN models, LSTMs can learn dependencies over longer intervals more efficiently [42]. In this study, CLOUT represented all LSTM-based predictive models we built for EHRs. The central architecture, as shown in Figure 1, is an attention-based LSTM model that processed the encounter vectors and made a binary class prediction.

Given a patient with encounters, the encounter vectors derived from a CLOUT model are *e*_1_, *e*_2_, ... *e*_n_. We ran the encounter vectors through the LSTM framework to get the hidden vectors at each time step, *h*_1_, *h*_2_, ... *h*_n_. We then used the attention module to find the weighted sum of these hidden vectors . Formally, *H* = *a*_1_ . *h*_1_ + *a*_2_ . *h*_2_ + ... *a_n_* . *h_n_*. The vector was then sent through a linear layer, and the output was squashed between 0 and 1 using a sigmoid function. This final output represented the probability of a positive class, which in our current application was the probability that the patient died.

Note that our LSTM architecture is commonly used for sequence data. The innovation of this work is the representation of the encounter vector that integrates different types of EHR data, which we will describe below.

#### A Simple Concatenation Model

In this version of CLOUT, the encounter vector was derived by a simple concatenation of different types of features. Every patient encounter had a set of documented International Classification of Diseases (ICD) codes, medications, and laboratory components. We converted these to 3 bit-vectors, , , and , respectively, each of the size of the vocabularies. Bit-vectors are vectors of size equal to the length of the vocabulary with 1 at the index where the feature is documented and 0 everywhere else. We passed these bit-vectors through linear embedding layers to get their dense vector representations. We concatenated these dense vector representations and passed the resultant vector through a nonlinear function such as the rectified linear unit [43] to get the final encounter representation, .

#### Representation Through Concatenation With Autoencoders

Recent work on word embeddings called ELMo [44] has shown that integrating different levels of representations learned by neural networks improves predictive performance in natural language processing applications, as different layers represent different characteristics of input data. Building on the same concept, we created a CLOUT model that integrates the representations of input features learned from an autoencoder with our inputs before sending them through the prediction layer. The hidden layer representations contain valuable information about the relationships between different input features, and by including this information along with the actual input features, we enable the model to make predictions with more knowledge. We integrate the representations using concatenation.

#### The Latent Space Representation

**Figure 2:**
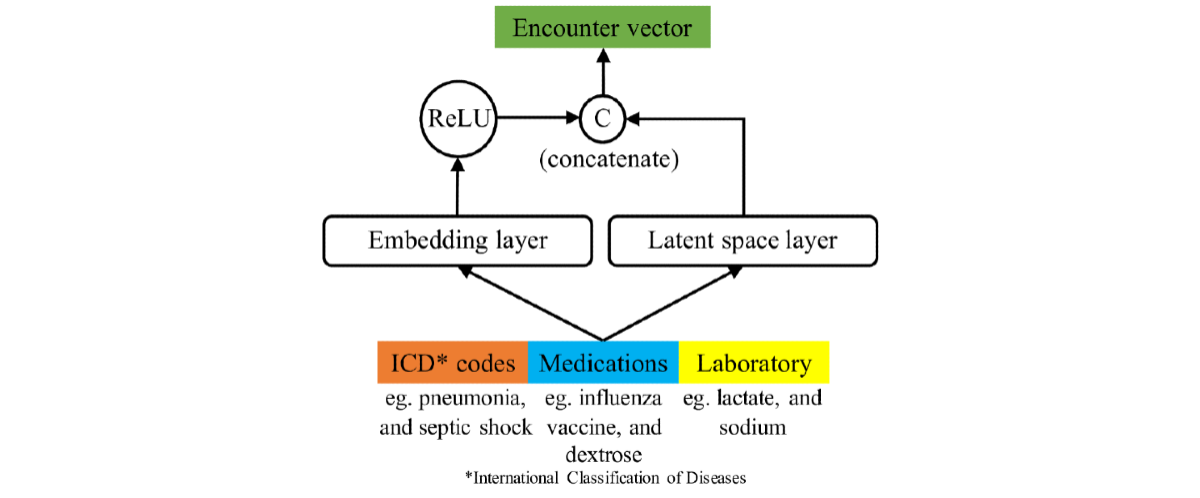
Model for constructing the encounter vector. ReLU: rectified linear unit; ICD: International Classification of Diseases.

ICD codes, medications, and laboratory results are not isolated unrelated clinical information. They are clinically intertwined or correlated. For example, as stated earlier, medications depend upon the diagnoses of the patient in that encounter. To capture the correlations among EHR data, we added a multi-view latent space component, as shown in Figure 2, by adapting a correlational neural network [45] framework.

**Figure 3:**
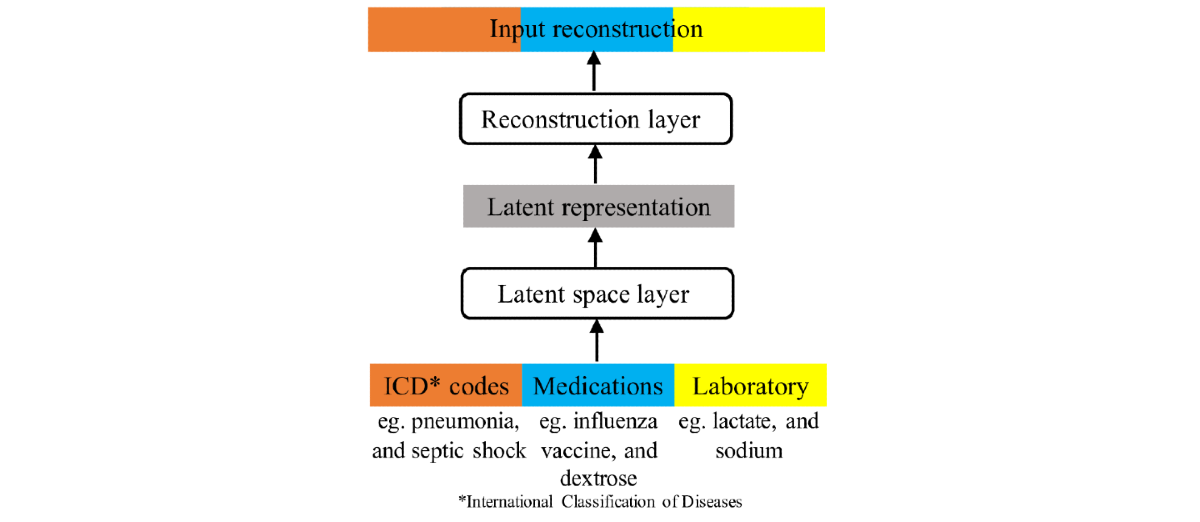
The correlational neural network for our 3 views. ICD: International Classification of Diseases.

We used a correlational neural network for 3 views (ICD codes, medications, and laboratory components) to construct the latent representation for our latent space model. This component is graphically shown in Figure 3.

The latent space representation is a measure of the patient condition—a combination of related information from diagnosis codes, medications, and laboratory components. The details of this component are further described in Multimedia Appendix 3.

To integrate latent space representation into the encounter vector, we first projected the encounter into this latent space to get the latent space vector, *l*. We simultaneously performed all of the operations in the simple concatenation version to find the encounter vector of that version, *e_c_*. The final encounter vector was the concatenation of *l* and *e_c_*. The model described here is shown in Figure 2. Note that the c-operation stands for concatenation.

To evaluate the effectiveness of the correlational neural network, we also implemented a traditional autoencoder with one hidden layer *f* and one output layer *g* with the goal to reconstruct the input using a hidden representation of lower dimensions. We called this model CLOUT-autoencoder**.**

#### Evaluation

We evaluated each of the baseline and CLOUT models on the p-MIMIC dataset. We obtain true-positives (TP), false-positives (FP), true-negatives (TN), and false-negatives (FN). We report area under the receiver operating characteristic curve (AUC-ROC) scores for all models, and precision 
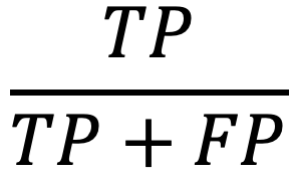
, recall 
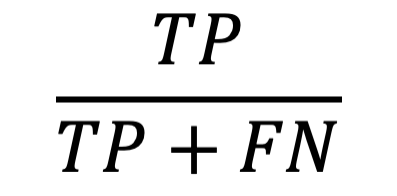
, and F1-scores 
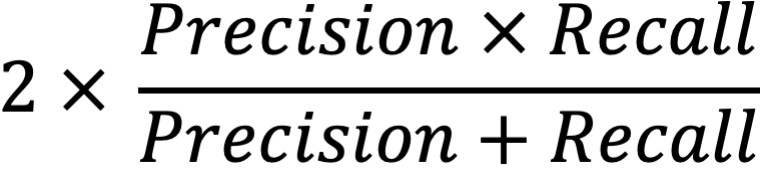
 for the top performing models.

### Risk Factor Experiment With Physicians

Predictive models would be of limited clinical use if the models are not interpretable. To interpret or identify the risk factors in our CLOUT models, we conduct an ablation experiment, which has been widely used for feature engineering. We perturb the patient data to zero out the contribution of a feature and calculate the corresponding difference in output. This classical method shows the contribution of each feature, which may correspond to the risk score.

Recall that each of our CLOUT models outputs a probability score that indicates mortality risk. So, the difference in output would be the reduction in this probability, which we call the attribution weight of the given feature. We calculated the attribution weight for each ICD code, medication, and laboratory component that is documented in the patient's EHRs. These features would then constitute the risk factors associated with the mortality, and the attribution weight represents the strength of the association.

Although ablation experiments have been widely used for feature engineering and interpretation of neural network models in many applications [46], they have not been evaluated for identifying risk factors of patient outcome based on longitudinal EHRs.

Therefore, we designed a comprehensive evaluation of the risk factors ranked by CLOUT and compared them with ones ranked by a logistic regression model. Specifically, we ranked the risk factors at the patient level and population level. At the patient level, each risk factor (ie, feature or variable) is weighted by its contribution to the correct prediction to the patient. We ranked the risk factors at the population level by aggregating and normalizing the attribution weights of features across the patient population.

#### Experiment Design

Using stratified random sampling, we selected a subset of risk factors from the prediction models CLOUT and logistic regression, respectively, and asked 5 unbiased physicians (4 internists and 1 cardiologist), who were not privy to the reasons for doing the ranking, to independently judge the clinical relevance of those risk factors.

To reduce the total number of features that the physicians need to evaluate, we selected features from CLOUT. Specifically, for each feature, the ablation experiment output a relevance score. We bin the features into 3 groups: (1) top 20 features, (2) 20-50 features, and (3) the remaining features. From each bin, we randomly selected 4 features. We then randomly selected 1 patient and accordingly obtained a total of 12 features for that patient. We also obtained the ranked list of features by population and followed a similar bin strategy to select another 18 features distributed across the different feature sets (we purposely selected those features that differ from the features we selected from the sample patient so that we could maximize our evaluation features). Therefore, we selected a total of 30 features (12 by a patient and 18 by the population).

We randomized those 30 features and asked the 5 physicians who are blinded to the CLOUT rankings to evaluate, for each feature, its clinical relevance. Specifically, we asked each physician to score the feature (1-5, with 1 as the least relevant and 5 the most relevant) based on their clinical knowledge or guidelines.

We calculated the Pearson correlation coefficient between physicians’ scores for pairwise agreements between physicians, and between the CLOUT scores and physicians’ scores. We also performed a *t* test for statistical significance. We used the same 30 features to evaluate the logistic regression model and, in this case, using the weight assigned by the logistic regression model for the ranking.

Finally, we performed another evaluation where we first averaged the scores of all the physicians to obtain a representative gold standard. We then computed the correlation coefficients between these scores and the scores from our models and the logistic regression baseline.

## Results

### Model Performance

During our experiments, we found that models using abnormal laboratory components as input (ie, binary coding of normal/abnormal) performed better than those using all the laboratory components. Therefore, the results presented here for the p-MIMIC dataset used only the abnormal labs recorded in patient encounters through an abnormal flag.

**Figure 4:**
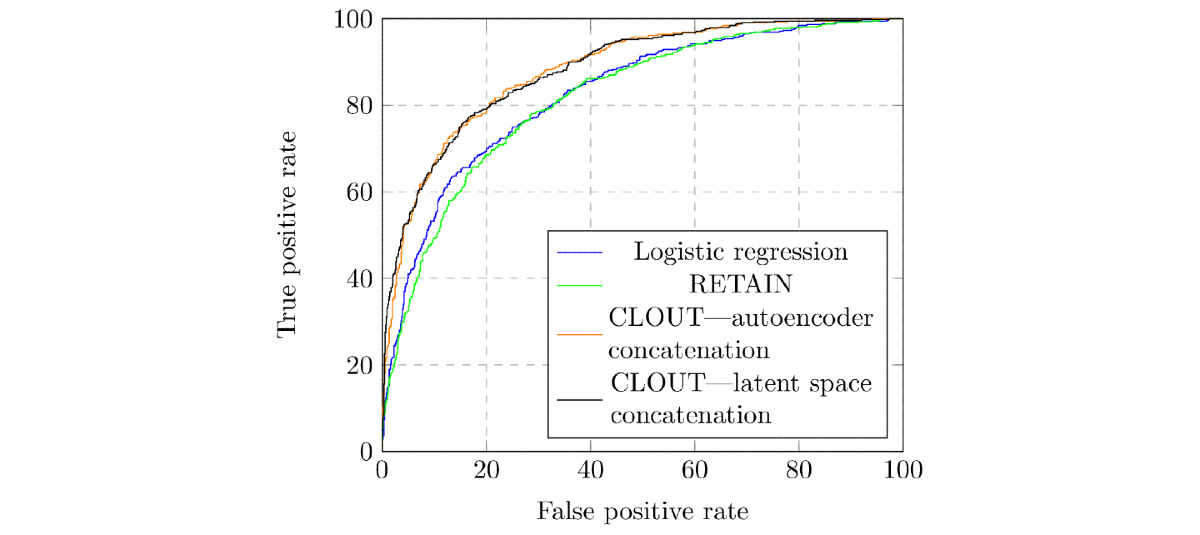
The area under the receiver operating characteristic curves for various models. RETAIN: Reverse Time Attention model; CLOUT: L(STM) Outcome prediction using Comprehensive feature relations.

As shown in Table 2, the AUC-ROC results for our CLOUT models are significantly better (*P*<.001) than both the RETAIN and the logistic regression models. The AUC-ROC curves for the representative models are presented in Figure 4. Our CLOUT model with concatenated latent space representation (Figure 2) achieved 0.89 AUC-ROC score, which is more than 0.06 absolute increase over the ICD-RETAIN, logistic regression, and simple LSTM models and a 0.02 increase over RETAIN with all codes. To get a better understanding of our results, we also present the precision and recall scores for each class for the top models in Table 3.

Our latent space representation model also slightly outperformed the traditional autoencoder CLOUT model, although it is not statistically significant. An important result here is the integration of different levels of representations (input space and from either an autoencoder or a correlational autoencoder) substantially improves the performance of a model, which outperforms one that uses autoencoder alone. The code for our models and experiments can be found at our CLOUT repository [47].

**Table 2:** Area under the receiver operating characteristic curve scores for different models.

Method	Area under the receiver operating characteristic curve, mean (SD)
Logistic regression	0.82 (0.0103)
RETAIN^a^ (only ICD^b^)	0.82 (0.0924)
TaRETAIN^c^-*first* (only ICD)	0.82 (0.0118)
TaRETAIN-*prev* (only ICD)	0.82 (0.0919)
RETAIN (all codes)	0.86 (0.0105)
Long short-term memory with only ICD codes	0.83 (0.0104)
CLOUT^d^—only autoencoder	0.80 (0.0116)
CLOUT—only latent space	0.81 (0.0082)
CLOUT—simple concatenation	0.88 (0.0096)
CLOUT—autoencoder concatenation	0.88 (0.0107)
CLOUT—latent space concatenation	*0.89 (0.0138)* ^e^

^a^RETAIN: Reverse Time Attention model.

^b^ICD: International Classification of Diseases.

^c^TaRETAIN: time-aware RETAIN.

^d^CLOUT: L(STM) Outcome prediction using Comprehensive features relations.

^e^Best performing model.

**Table 3:** Precision, recall, and F-scores for top CLOUT^a^ models.

Method and class		Precision	Recall	F-score
**CLOUT—Simple concatenation**				
	0	0.85	0.82	0.83
	1	0.71	0.76	0.73
	Average	0.80	0.79	0.80
**CLOUT—Autoencoder concatenation**				
	0	0.85	0.85	0.85
	1	0.74	0.74	0.74
	Average	0.81	0.81	0.81
**CLOUT** **—** **Latent space concatenation**				
	0	0.84	0.88	0.86
	1	0.78	0.72	0.72
	Average	0.82	0.82	0.82

^a^CLOUT: L(STM) Outcome prediction using Comprehensive features relations.

### Risk Factors

To measure agreements among physicians, we compute the Pearson correlation coefficient between their scores. For patient-specific features, Table 4 shows the Pearson correlation coefficient between each pair of physicians and also between different models and the physicians. With the physician gold standard ratings computed by averaging, we found that our model had a correlation coefficient of 0.64, which is higher (4.9%) than the correlation coefficient of 0.61 with the logistic regression model.

**Table 4:** Pearson correlation coefficients for agreement between physicians and models.

Agreement		Physician 1, *r*		Physician 2, *r*		Physician 3, *r*		Physician 4, *r*		Physician 5, *r*		Mean (SD)	
**Physician-physician agreement**													
	Physician 1		1.00		0.81		0.56		0.61		0.88		0.72 (0.13)
	Physician 2		0.81		1.00		0.87		0.65		0.86		0.80 (0.09)
	Physician 3		0.56		0.87		1.00		0.49		0.69		0.65 (0.14)
	Physician 4		0.61		0.65		0.49		1.00		0.61		0.59 (0.06)
	Physician 5		0.88		0.86		0.69		0.61		1.00		0.76 (0.11)
**Physician-model agreement**													
	Logistic regression		0.60		0.63		0.53		0.32		0.52		0.52 (0.11)
	RETAIN^a^		0.65		0.72		0.61		0.30		0.58		0.57 (0.14)
	CLOUT^b^—only autoencoder		−0.07		0.13		0.21		*0.* *55^c^*		0.17		0.20 (0.20)
	CLOUT—only latent space		0.42		0.77		*0.* *64*		0.35		0.53		0.54 (0.15)
	CLOUT—simple concatenation		0.52		0.64		0.70		0.19		*0.* *67*		0.54 (0.19)
	CLOUT—autoencoder concatenation		0.54		0.70		0.64		0.14		0.62		0.53 (0.20)
	CLOUT—latent space concatenation		*0.* *69*		*0.* *77*		0.59		0.18		*0.* *67*		*0.58 (* *0.21* *)*

^a^RETAIN: Reverse Time Attention model.

^b^CLOUT: L(STM) Outcome prediction using Comprehensive features relations.

^c^Italicization signifies highest physician-model agreement in the column.

## Discussion

### Principal Findings

In this study, we have developed innovative CLOUT models and compared them with other state-of-the-art predictive models with respect to performance on mortality prediction. We found that the performance of almost every CLOUT model surpassed the competitive baseline models (eg, RETAIN). The results support that LSTM is a state-of-the-art framework for EHR-based predictive modeling.

Our results showed that the integration of different levels of latent representations (input space and from either an autoencoder or a correlational autoencoder) substantially improves the performance from 0.80 to 0.88 AUC-ROC. The rich representation may provide extra information to the model, which in turn helps the model make better predictions. The integration of different types of features (ie, ICD codes, laboratories, and medications) however had a mixed result. Specifically, the CLOUT model that incorporated only the abnormal laboratory results slightly surpassed the CLOUT model that incorporated all 3 features. This supported the importance of laboratory results for predicting mortality. Our results also suggested that there may be noisy information in the features. When CLOUT was implemented with the latent vectors included, it had the highest performance, an AUC-ROC score of 0.89 and an F1 score of 0.82. The result supports our approach of using the correlational neural network to identify latent vectors to best represent different but related clinical observations or variables.

On the other hand, when we incorporated temporal information as a feature, we showed little improvement in performance using RETAIN. A possible future direction is to explore time-dependent attentions, which may allow the model to integrate the temporal information in the architecture.

For the risk factors identified by our models, the average correlation coefficient between the physicians is mean 0.71 (SD 0.13), and the average Pearson correlation coefficients between CLOUT and the physicians and between logistic regression and physicians were 0.58 (SD 0.21) and 0.52 (SD 0.11), respectively. These results show a significant difference between the agreement among physicians and the agreement between the logistic regression model and the physicians (*P*=.04). In contrast, the difference in agreement between the CLOUT models and physicians is not statistically significant, strongly supporting the validity of risk factors and their ranking identified by CLOUT.

We also calculated the agreement with RETAIN for reference, and we found that the average was 0.57 (SD 0.14), which is still slightly less than the CLOUT model, with CLOUT losing out a lot with physician 4. Other CLOUT models also have slightly lower scores as reported in Table 4, but it is notable that the latent vector models that use the correlational autoencoder have better correlations (0.58, SD 0.21) with physicians than the ones that use a simple autoencoder (0.53, SD 0.20). The evaluation with our gold standard (the average physician scores) also informs us that CLOUT selects more meaningful features.

Our results show that physician 4 had a low correlation score with other physicians as well as with our CLOUT models. For example, lactulose enema, and encephalopathy not otherwise specified were scored as 2 by physician 4, whereas all the other physicians gave scores of 4 or greater. When we removed physician 4, the correlation between the latent space CLOUT model and the physicians improved from 0.58 to 0.68.

For population-level features, we performed similar evaluation between physician scores and the CLOUT model scores, and the average correlation coefficient values were ICD codes −0.19, medications −0.43, and laboratory components −0.37, which are lower than patient-specific interpretations. This is not surprising as many risk factors (eg, severe diseases) are rare events that are not present for patients in general.

Furthermore, CLOUT models captured important risk factors while making predictions. In general, our CLOUT models show that patients with diagnosis codes representing cranial nerve disorder and cystic liver disease were marked with a high risk of mortality. This is reasonable as those are diseases with a high risk of mortality.

### Limitations

Our dataset was constructed from EHR data and is, hence, prone to standard data quality issues that EHRs typically have, as documented in the literature. EHRs are known to have missing diagnoses and medication codes for patients when compared with insurance claims. Furthermore, our analysis of ICU admissions does not account for death because of accidental circumstances such as car crashes. We used all the information exactly as it appears as it is infeasible to comb through all the records to pick patients for the study. Another limitation we would like to report is the absence of vital sign features in our dataset, which we ignore because of the involved preprocessing steps that are required to handle missing numerical values.

The CLOUT models have significant limitations as well. First, similar to most predictive models, the risk factors identified by the CLOUT models include cofounding variables. For example, we found that patients who have a prescription for a scopolamine patch have high-risk scores. This is a medication prescribed to terminally ill patients as part of palliative care regimen to reduce excessive airway secretion. So, in this case, the actual reason for palliative care is a strong risk factor for death, not the medication, which is a confounding factor. Another limitation of our work is that our models are very dependent on the population size. Bias could be introduced when the size is small. However, such limitations exist in most predictive models not reviewed or guided by physician oversight.

### Comparison With Prior Work

We surveyed a variety of approaches to compare our models. This includes statistical approaches [48-52], deep learning–based approaches [6,38,53-55], and other phenotyping efforts [4,5]. We also surveyed papers on interpretability. A detailed analysis of all this can be found in Multimedia Appendix 4.

### Conclusions

EHRs are widely available and have enormous untapped potential to predict patients’ health outcome. EHR-based predictive models are potentially hugely useful for clinical decision support. Our experiments show that incorporating comprehensive clinical information is useful and can improve predictions and that integrating latent space representations learned through a correlational neural network to clinical information led to the best performing CLOUT model. Our risk factor experiment with physicians also suggests that CLOUT models find more clinically relevant risk factors. Our results strongly support that CLOUT may be a useful tool to generate clinical prediction models, especially among hospitalized and critically ill patient populations.

The future directions include new models to incorporate the temporal information and methods to integrate clinical notes for predictive models. We may also explore other models to integrate different views, including the Capsule network model [56].

## Appendix

Multimedia Appendix 1 The Medical Information Mart for Intensive Care-III, preprocessing, and outcome label.

Multimedia Appendix 2 Relevant machine learning components.

Multimedia Appendix 3 Correlational neural network.

Multimedia Appendix 4 Comparison with prior work.

## Abbreviations

CNN: convolutional neural network
CLOUT: L(STM) Outcome prediction using Comprehensive features relations
EHR: electronic heath record
ICD: International Classification of Diseases
ICU: intensive care unit
LSTM: long short-term memory
MIMIC-III: Medical Information Mart for Intensive Care-III
RETAIN: Reverse Time Attention model
RNN: recurrent neural network
TaRETAIN: time-aware RETAIN
