# Supplementary material for: Learning Latent Space Representations to Predict Patient Outcomes: Model Development and Validation
Source: J Med Internet Res. 2020 Mar 23;22(3):e16374. doi: 10.2196/16374 (PMC7136840; doi:10.2196/16374)
Supplement: Multimedia Appendix 1 [file jmir_v22i3e16374_app1.docx]

**Appendix 1 – The MIMIC-III Dataset, Pre-processing, and Outcome Label**

All models are trained and evaluated on the MIMIC-III (Medical Information Mart for Intensive Care) dataset, an EHR dataset made publicly available by the MIT Lab for Computational Physiology. The dataset contains 7,537 patients with 2 or more encounters, which we used to build our CLOUT and baseline models. The demographic information for patients in this dataset is given in Table 4. Note that an encounter represents a single visit or admission of the patient to the hospital Intensive Care Unit (ICU). The maximum numbers of encounters documented for a patient is 42, and the average is 2.65 $\pm$ 1.62.

Table 4. Patient Demographic information

| **Age** | |
| --- | --- |
| Mean | 74.74 |
| Median | 66.00 |
| **Sex** | |
| Male | 56% |
| Female | 44% |

| **Race** | |
| --- | --- |
| White | 75% |
| Black | 12% |
| Hispanic | 4% |
| Asian | 3% |
| Other/unknown | 6% |

Each encounter contains the diagnosis codes, laboratory tests and their results, and prescribed medications documented in that encounter. Notes are also available, although we did not incorporate free-text notes in the current CLOUT model. We found a significant amount of duplication due to components being documented multiple times for each admission encounter, which we pre-processed and removed.

There are 27,854,055 component laboratory tests documented in MIMIC III. We found that around 5,609,021 (20%) of the these did not have associated encounters, so we removed them and relied upon the remaining 22,245,034 for our predictive models. For the laboratory tests, we ignored the actual component values and used only their abnormal flags as the features (i.e., binary coding). Table 5 shows statistics regarding the number of ICD codes, labs, and medications for an encounter.

Table 5. Distribution of clinical features per encounter.

| **Type** | **Min** | **Max** | **Mean (Std)** |
| --- | --- | --- | --- |
| ICD codes | 1 | 39 | 11.93 $\pm$ 5.98 |
| Medications | 0 | 164 | 33.64 $\pm$ 20.04 |
| Laboratory components | 0 | 107 | 27.18 $\pm$ 13.62 |

We converted the structured data into binary vectors of the size of the vocabulary, which is the total numbers of distinct ICD codes, labs, and medications recorded. After pre-processing the MIMIC-III dataset, the data we used in this study included 7,537 patients, with a total of 942 different ICD codes, 3,202 different medications, 681 different laboratory tests, and 284 different abnormal laboratory tests. We call this pre-processed dataset p-MIMIC.

The outcome label we used in this study is the patient mortality, which has been used as the outcome in other works [5], which we have included as a baseline model. This label is obtained in the MIMIC dataset from the hospital records and the social security death records. This indicates the death of a patient after the final encounter, which we want to predict using patient's longitudinal EHRs.
