# Supplementary material for: Learning Latent Space Representations to Predict Patient Outcomes: Model Development and Validation
Source: J Med Internet Res. 2020 Mar 23;22(3):e16374. doi: 10.2196/16374 (PMC7136840; doi:10.2196/16374)
Supplement: Multimedia Appendix 2 [file jmir_v22i3e16374_app2.docx]

**Appendix 2 – Relevant Machine Learning Components**

The CLOUT models are built upon the state-of-the-art LSTM framework. Here, we provide a description of relevant concepts or components that are built into our CLOUT models.

**Bit Vector**

A bit vector is a standard representation used for an input instance with multiple documented features. It is a vector of a fixed size where the element at each index is either a 1 or a 0 depending on whether the feature corresponding to that index has been documented. For example, if a patient has 5 distinct ICD codes documented out of a total possible 100 ICD codes, the bit vector would be of size 100 with 5 elements set to 1. The indices of these five elements is based on which of the 100 features were documented.

**Embedding layer**

One-hot vectors are typically sparse. They are rarely used in neural network models like RNNs or CNNs directly. Instead they have to be converted into a dense vector of a smaller dimension size that captures the information present in the one-hot vector. This is done by an embedding layer. An embedding layer simply performs a linear transformation on the one-hot vector to get another vector of the desired size. Mathematically, given a one-hot vector $o$ of size $m$ and a target dense vector size $n$, an embedding layer consists of a set of weights $W$of size $m\times n$ and biases $B$ of size $n$. The dense vector $d$ we get from this embedding layer is given by

$$d=W.o+B$$

**RNN and LSTM**

RNN is a class of artificial neural network where connections between nodes form a directed graph along a sequence. For this work, we have patients with EHRs that consist of information documented over multiple admission encounters. Here, RNNs learn a mathematical representation of the evolving state of the patient over time and we can use this longitudinal representation to make predictions. LSTM networks are a special kind of RNNs that perform well when the number of time steps is high by learning long-term dependencies.

**Attention mechanism**

This is a modification proposed to RNNs to capture the idea that information in some time steps is more important than those in others. Typically, this mechanism learns a set of attention weights $a_{1}\ldots a_{n}$ by which the information in respective timestamps $t_{1}\ldots t_{n}$ is weighted.

**Autoencoders**

Autoencoders are multi-layer neural networks that aim to learn a high-level or hidden representation of input vectors using unlabeled data. Typically, the size of this hidden vector is lower than the size of the input vector (dimensionality reduction). Exact details are in the Appendix 3 but the idea behind these models is to learn weights to reconstruct the input vector using just the hidden vector.

**Correlational Neural Network**

A correlational neural network is a special kind of autoencoder. This kind of a model is used to learn high-level representations of input vectors that contain multiple feature sets of correlated data. For example, in our case, patients have both diagnoses features as well as medication features. This network is designed to learn the high-level representation by accounting for the correlations between the different sets of data. By doing this, this network captures rich representation across feature sets than a simple auto-encoder. The full details of this network are present in the Appendix 3.
