# Supplementary material for: Learning Latent Space Representations to Predict Patient Outcomes: Model Development and Validation
Source: J Med Internet Res. 2020 Mar 23;22(3):e16374. doi: 10.2196/16374 (PMC7136840; doi:10.2196/16374)
Supplement: Multimedia Appendix 3 [file jmir_v22i3e16374_app3.docx]

**Appendix 3 – Correlational Neural Network**

Let us formally define a set of data instances $Z$ with two parallel views $X$ and $Y$; a single instance $z_{i}$ can be seen as $(x_{i}, y_{i})$. In the medical context, $Z$ would be the set of patients, and $X$ and $Y$ would be one of the feature sets like diagnoses, medications, or laboratory components.

If $X$ and $Y$ are highly dependent on each other, we should be able to construct one view given the other. This means there is some latent space where the projections of instances from both views would be the same or very similar. We want to find this latent space since it captures important cross-view dependencies. In the medical context, we can think of this latent space as some sort of patient wellness space. To find the patient's diagnoses, we would simply project the patient's representation in this space to the diagnoses space and likewise for medications.

A correlational neural network is an auto-encoder with a modified loss function, which can be used to find such a latent space. In a traditional auto-encoder with one hidden layer $f$ and one output layer $g$, we try to reconstruct the input using a hidden representation of lower dimensions. Given an input vector $z_{i}$, let $f(z_{i})$ and $g(f(z_{i}))$ be the hidden and output vectors from the forward pass in the autoencoder. The model is trained using the reconstruction loss $\mathcal{R}$ between the input and output as the loss function. The loss function of a traditional auto-encoder is hence given by

$$\mathcal{L}_{traditional}= \sum_{i=1}^{N} \mathcal{R (}z_{i}, g(f\left( z_{i} \right)))$$

In a correlational neural network, the loss function is modified to include three new terms, apart from the standard auto-encoder reconstruction loss. The first new term is the reconstruction loss if only the data from the first view is used to reconstruct the entire input. The second term is the same metric for using only data from the second view. The final term is an adjusted correlation coefficient which is used to reward the model for learning hidden representations of the views that are correlated. The loss function of a correlational neural network is therefore given by

$$\mathcal{L}_{cornn}= \sum_{i=1}^{N} \mathcal{R}\left( z_{i}, g\left( f\left( z_{i} \right) \right) \right)\mathcal{+ R (}{(x}_{i,},0), g(f\left( z_{i} \right)\mathcal{))+ R (}{(0, y}_{i}), g(f\left( z_{i} \right)))- \alpha cor(f(x_{i},0), {f(0, y}_{i})$$

To adapt this to three or more views, we extended the reconstruction loss terms to reconstruct the input using just one input view each time. We use a value $\varsigma$ to identify the correlation among the multiple views, given by

$$\varsigma= \frac{\kappa(x_{i}, y_{i},u_{i},\ldots)}{\sqrt{\vartheta\left( x_{i} \right).\vartheta\left( y_{i} \right).\vartheta\left( u_{i} \right)\ldots}}$$

where $\kappa$ is the joint cumulant of the given variables and $\vartheta$ is the variance. $x, y, u$ represent different parallel and dependent views of data. We can think of the joint cumulant as a substitute for the correlation coefficient for more than two views. This idea was proposed in [56].
