# Supplementary material for: Learning Latent Space Representations to Predict Patient Outcomes: Model Development and Validation
Source: J Med Internet Res. 2020 Mar 23;22(3):e16374. doi: 10.2196/16374 (PMC7136840; doi:10.2196/16374)
Supplement: Multimedia Appendix 4 [file jmir_v22i3e16374_app4.docx]

**Appendix 4 – Comparison with Prior Work**

***Predictive modeling using statistical approaches***

Predictive modeling approaches have been developed to use EHR data to predict various clinical events. Traditional methods like logistic regression and decision trees have been used in the past to create such models, with notable successes [48]–[52]. These models tend to be readily interpretable by end users due to their inherent simplicity and reliance on pre-specified hypotheses.

***Predictive modeling using deep learning***

More recently, neural networks with deep architectures are being increasingly employed for clinical predictions. A notable relevant work is the model called RETAIN (REverse Time AttentIoN) [6]. However, RETAIN only makes use of the ICD-codes for predictions, ignoring medications and laboratory tests in an attention-based RNN setting. Dipole [53] is a more recent effort that uses bi-directional RNNs with multiple attention mechanisms for interpretability. Health-ATM [54] uses a hybrid convolutional-recurrent neural network architecture with target aware attentions to further the accuracy scores. Despite more complex architectures, all aforementioned models either use a single feature set or concatenated feature sets, without modeling any latent spaces or relations between different types of feature sets.

A more recent notable work in this field deals with scalability in a large EHR datasets [38]. This work is based on a deep neural network architecture and reported a high AUC-ROC score range around 0.93-0.94 for in-hospital mortality. The work however uses a much larger and more comprehensive dataset with detailed time stamps and feature values than the MIMIC-III dataset. The work integrated several hand-engineered features but no latent representation. Author and colleagues [55] provided a good overview of the predictive modeling research with the EHR data using deep learning, and the challenges involved.

***Patient Phenotyping***

In machine learning, patient phenotyping refers to the process of constructing meaningful patient representations from raw EHR data. Deep Patient [4] is one of the well-known models in this field. Deep Patient uses multiple layers of autoencoders to build a high-level representation of patient data. The Deep Patient approach considers multiple types of the EHR data, including ICD codes, procedures, and laboratory tests, but it doesn't explicitly model the relations among the data. In contrast, our approach in this paper was to learn a latent space representation or complex relations between different types of EHR data, which we believe to account for our improved results. Moreover, Deep Patient only uses the representation of autoencoders [4]. Recent work [44] has shown that integrating different levels of representations learned by neural networks further improve natural language processing applications. In this work, we found that integrating the low-level word embedding with representations learned by autoencoders significantly improve CLOUT. Another relevant work is data-driven phenotyping [5], which only uses structured diagnosis codes - like the DxGroups or HCC codes, but this work does not use comprehensive, multi-featured data.

***Interpretability***

Due to the inherent non-linearity in NN architectures, it is difficult for many end users to interpret the NN models. Making NN architectures interpretable is an active research in machine learning. To this end, recurrent neural networks were fitted with attention mechanisms that associate input features with attention scores in making a prediction. These scores can be used to interpret which features were important in making a decision and the mechanism was shown to work well in some applications [56], [57]. RETAIN and Health-ATM models also use variants of this attention to make interpretable predictions. However, the attention mechanism comes at the cost of performance due to added complexity in the model. In this study, we use a different approach that has been widely used for feature engineering for interpretability that works with any model without interfering with the model’s structure. We also provide the first evaluation of the identified risk factors by NN models.
